# Diminished Memory T-Cell Expansion Due to Delayed Kinetics of Antigen Expression by Lentivectors

**DOI:** 10.1371/journal.pone.0066488

**Published:** 2013-06-18

**Authors:** Karina Furmanov, Mazal Elnekave, Abdallah Sa'eed, Hadas Segev, Luba Eli-Berchoer, Darrell N. Kotton, Gilad Bachrach, Avi-Hai Hovav

**Affiliations:** 1 Institute of Dental Sciences, Hebrew University-Hadassah School of Dental Medicine, Jerusalem, Israel; 2 The Pulmonary Center and the Department of Medicine, Boston University School of Medicine, Boston, Massachusetts, United States of America; Federal University of São Paulo, Brazil

## Abstract

Memory CD8^+^ T lymphocytes play a central role in protective immunity. In attempt to increase the frequencies of memory CD8^+^ T cells, repeated immunizations with viral vectors are regularly explored. Lentivectors have emerged as a powerful vaccine modality with relatively low pre-existing and anti-vector immunity, thus, thought to be ideal for boosting memory T cells. Nevertheless, we found that lentivectors elicited diminished secondary T-cell responses that did not exceed those obtained by priming. This was not due to the presence of anti-vector immunity, as limited secondary responses were also observed following heterologous prime-boost immunizations. By dissecting the mechanisms involved in this process, we demonstrate that lentivectors trigger exceptionally slow kinetics of antigen expression, while optimal activation of lentivector-induced T cells relays on durable expression of the antigen. These qualities hamper secondary responses, since lentivector-encoded antigen is rapidly cleared by primary cytotoxic T cells that limit its presentation by dendritic cells. Indeed, blocking antigen clearance by cytotoxic T cells via FTY720 treatment, fully restored antigen presentation. Taken together, while low antigen expression is expected during secondary immunization with any vaccine vector, our results reveal that the intrinsic delayed expression kinetics of lentiviral-encoded antigen, further dampens secondary CD8^+^ T-cell expansion.

## Introduction

Since the protective capacity of memory CD8^+^ T cells is generally a function of their absolute number in the host, approaches to amplify their frequencies are constantly examined [1]. Viral vectors represent a powerful vaccine modality and numerous studies have demonstrated their ability to boost memory CD8^+^ T cells [2]. Viral vectors vary in their capacity to expand memory CD8^+^ T cells, partly, due to the presence of vector-specific immune responses [3]. However, such variations exist even in the absence of anti-vector immunity [4]. This suggests that vector-intrinsic features have a critical influence on their ability to boost cell-mediated immunity.

A successful boosting viral vector should have minimal pre-existing immunity, low anti-vector immunity and the potential to induce robust T-cell responses. Due to rare exposure to lentivirus, pre-existing immunity to lentiviral vectors (hereafter lentivectors) in the population is negligible [5]. In addition, vector-specific immune responses generated by lentivectors are relatively weak, since no viral proteins are expressed in the host during immunization, and host immunity is generated mainly against the pseudotyping envelope [6]. As for the immunogenicity of lentivectors, recent studies have shown their capacity to elicit robust and sustained T-cell responses that can protect against cancers and infectious diseases [7], [8], [9]. These imply that lentivectors could be an ideal vaccine modality to boost CD8^+^ T cells in a setting of heterologous prime-boost immunization. Moreover, it was thought that lentivectors can be used in multiple rounds of immunizations in order to augment “primary” immune responses as in the case of DNA vaccination [10].

Despite these attractive immunological traits, in this present study, we found that lentivectors elicited limited secondary T-cell responses following homologous and heterologous prime-boost immunizations. The magnitude of secondary CD8^+^ T cells failed to exceed those obtained by priming, even though considerable levels of antigen-specific CD8^+^ T cells were present in the mice at the time of boosting immunization. These results contrast with the conventional view that secondary T-cell responses should be superior to the primary response due to elevated frequencies of antigen-specific memory T cells in the primed host [11]. Indeed, we previously showed that viral vectors with a known strong anti-vector immunity, such as vaccinia and adenovectors, can induce potent secondary T-cell responses even in a setting of homologous prime-boost immunization [4]. It is thus likely that in addition to vector-specific immunity, lentivectors encompass unique qualities that interfere with their ability to boost efficiently memory CD8^+^ T cells. We therefore sought to dissect boosting immunization with lentivectors, as this will expand our understanding of the mechanisms regulating the generation of secondary T cells. This might also facilitate new strategies to improve the immunogenicity of lentivectors.

## Results

### Lentivectors Induce Limited Secondary CD8^+^ T cell Responses in the Absence of Anti-vector Immunity

**Figure 1:**
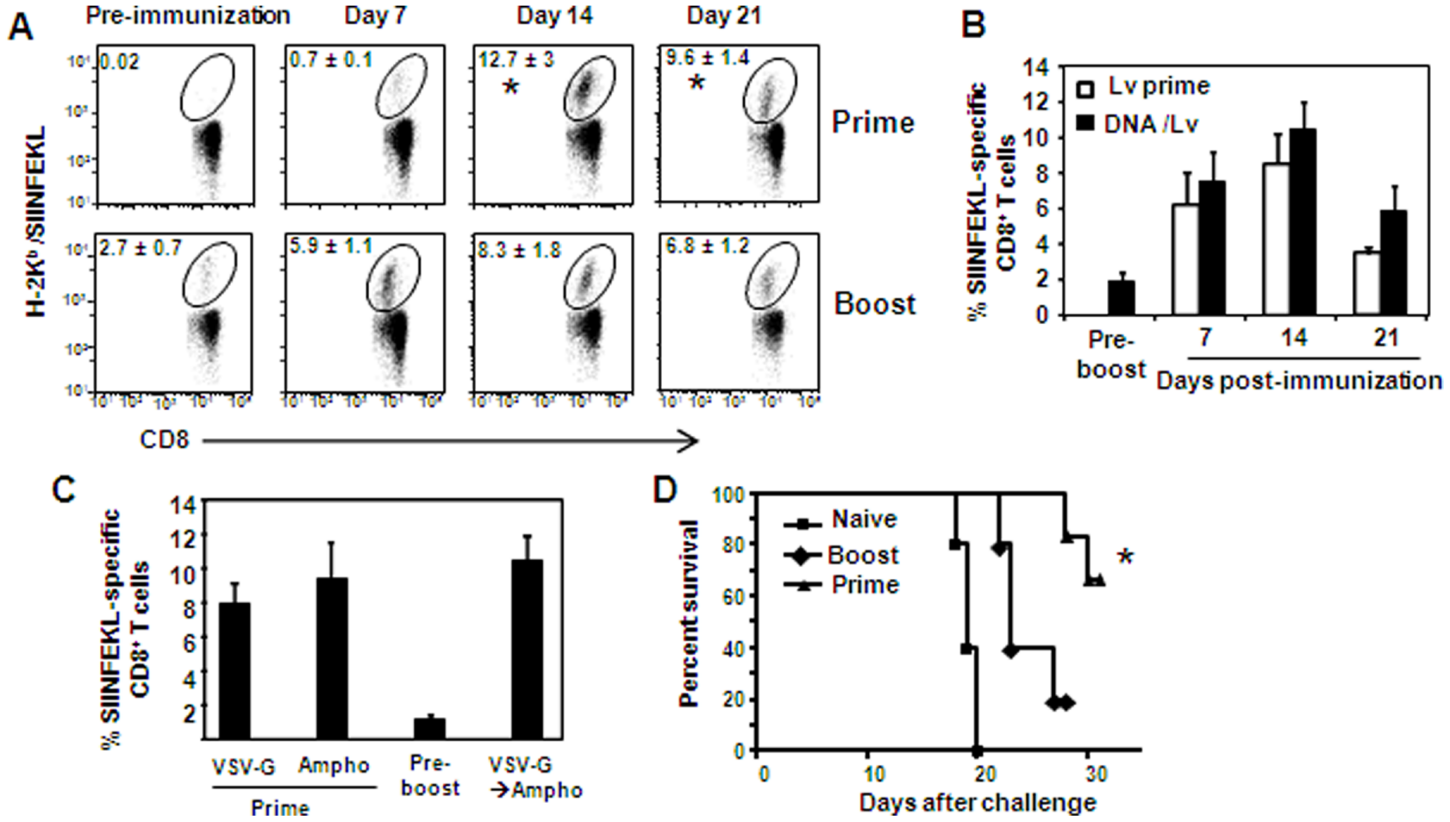
Limited expansion of secondary T cells following boosting with lentivectors. B6 mice were primed intradermally with 5×10^6^ TU of Lv-OVA, and seven weeks later the same mice, or another group of naïve B6 mice, were immunized via the same route and quantity of Lv-OVA to allow adequate comparison. (**A**) Representative flow plots display H-2K^b^/SIINFEKL tetramer-positive CD8^+^ T cells in the peripheral blood of primed versus boosted mice. Numbers indicate the frequencies of tetramar^+^CD8^+^ T cells and represent the mean of 5 mice per group ± SE. (**B**) Mice were primed with pACB-OVA plasmid and boosted seven weeks later with Lv-OVA (5×10^6^ TU) (DNA/Lv). Additional naïve mice were only primed with Lv-OVA (Lv prime). Antigen-specific CD8^+^ T cell responses were analyzed as described above. (C) Mice were primed intradermally with Lv-OVA containing the VSV-G envelope protein (VSV-G), and five months later mice were boosted with Lv-OVA expressing the Ampho envelope (VSV-GAmpho). For comparison, we simultaneously primed naïve mice with the Ampho expressing lentivectors (Ampho) and OVA-specific CD8^+^ T-cell responses were analyzed two weeks after immunization. (**D**) 15 days after priming or boosting with Lv-OVA each mouse was inoculated subcutaneously in the flank with 1×10^6^ B16-OVA cells and the survival kinetics were measured over time. Data represent the mean of 5–6 mice per group ± SE. *,*P*<0.05 primed mice versus boosted mice. The results depicted in this figure are representative of at least 2–4 independent experiments.

In order to examine the boosting capability of lentivectors, B6 mice were primed intradermally with lentivectors encoding the OVA antigen (Lv-OVA) (Fig. S1), and 7 weeks later the mice received a second immunization using the same route and vector quantity. As illustrated in Fig. 1A, despite the presence of OVA-specific CD8^+^ T cells in the primed mice, secondary immunization was not able to induce a robust expansion of these cells. In fact, the level of secondary CD8^+^ T cells was significantly lower than that obtained following primary immunization (*P*<0.005). To assess whether this low expansion of memory CD8^+^ T cells was due to vector-specific immunity, we used DNA vaccine for priming. DNA vaccines are successfully employed in numerous studies to prime CD8^+^ T cells for subsequent boosting with viral vectors [12]. Therefore, mice were primed with pACB-OVA plasmid (encoding the OVA antigen) and 7 weeks later boosted Lv-OVA. Nevertheless, lentivectors failed to efficiently boost plasmid DNA-elicited memory CD8^+^ T cells, in spite of the absence of anti-vector immunity (Fig. 1B). To further evaluate the issue of lentivector-specific immunity, we replaced the VSV-G envelope protein in our boosting vector with the envelope of the amphotropic murine leukemia virus (named Ampho), as it was shown that anti-vector immunity is generated mostly against the envelope protein [6]. Mice were also boosted five months after priming, to allow further T-cell differentiation into memory cells as compared to their effector-memory phenotype observed after 7 weeks (Fig. S2). We first showed that lentivectors harboring the Ampho envelope are very immunogenic, priming comparable levels of naïve CD8^+^ T cells to those achieved by VSV-G containing vectors (Fig. 1C). However, in agreement with our earlier observations, boosting mice with Ampho lentivectors failed to potently expand memory CD8^+^ T cells generated by VSV-G lentivectors, and the magnitude of these cells was similar to that obtained following priming (Fig. 1C). This demonstrates again the incapability of lentivectors to efficiently boost memory CD8^+^ T cells, even when the envelope protein was switched between the priming and boosting immunizations. We next assessed the functionality of secondary T cells by testing their ability to protect the immunized mice against a challenge with a lethal dose of B16 tumor cells expressing the OVA antigen (B16-OVA). As demonstrated in Fig. 1D, both immunized groups conferred protection in comparison to non-immunized mice (*P*<0.0005). In addition, superior survival kinetics were observed in primed mice as compared to boosted ones (*P* = 0.017). Collectively, our results demonstrated that lentivectors fail to boost efficiently the expansion of memory CD8^+^ T cells. Importantly, this incapability cannot be explained entirely by the presence of vector-specific immune responses.

### Truncated Antigen Presenting Activity Following Secondary Immunization with Lentivectors

**Figure 2:**
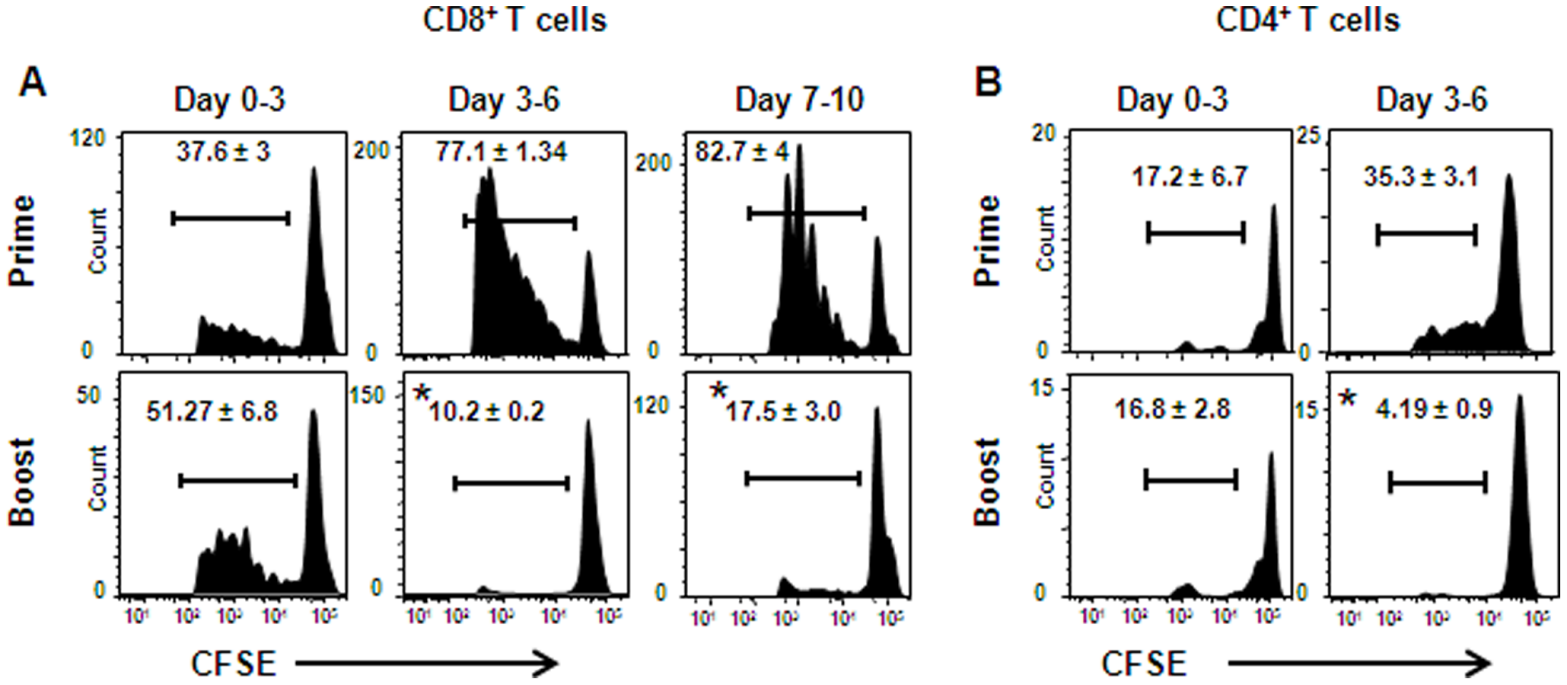
Shortened kinetics of antigen presentation *in vivo* in lentivector-boosted mice. B6 mice were primed, or primed and boosted with Lv-OVA, and then were adoptively transferred i.v. with 2×10^6^ CFSE-labeled OT-I (**A**) or OT-II (**B**) splenocytes at the indicated days. Three days later the LNs were harvested and the CFSE dilution was assessed by flow cytometry to analyze the proliferation of the transferred CD8^+^ or CD4^+^ T cells, respectively. Results are shown as representative flow plots gating on dividing CD8^+^ or CD4^+^ lymphocytes; numbers indicate the percentages of dividing cells and represent the mean of three mice per group for each time point ± SE. One representative out of 2 independent experiments is depicted. *, *P*<0.01, primed mice versus boosted mice at the time points indicated.

To further study T-cell induction by lentivectors, we examined the kinetics of T-cell activation *in vivo* in primed and boosted mice. B6 mice were primed, or primed and boosted with Lv-OVA, and on various times after immunization these mice were adoptively transferred with CFSE-labeled splenocytes purified from OT-I mice. Three days after each transfer LNs were collected and the CFSE-dilution in CD8^+^ OT-I cells was measured to determine their proliferative capacity. In Lv-OVA primed mice, moderate proliferation of OT-I cells was observed during the first 3 days post-immunization, and the proliferation increased considerably on the following days (Fig. 2A). Proliferation of OT-I cells in boosted mice was comparable to that seen in primed mice during the first 3 days post-immunization. However, this trend changed considerably, as CD8^+^ OT-I cells in the boosted mice proliferated poorly beyond the third day of immunization. Similar results were obtained following transfer of CFSE-labeled splenocytes purified from OT-II mice into Lv-OVA primed or boosted mice (Fig. 2B). These results demonstrate that secondary T cells experienced only a brief period of antigen stimulation *in vivo*, which may reflect their incapability to expand efficiently in boosted mice.

### Both Skin and LN-derived DCs Present Lentivector-encoded Antigen to Naïve and Memory CD8^+^ T cells

**Figure 3:**
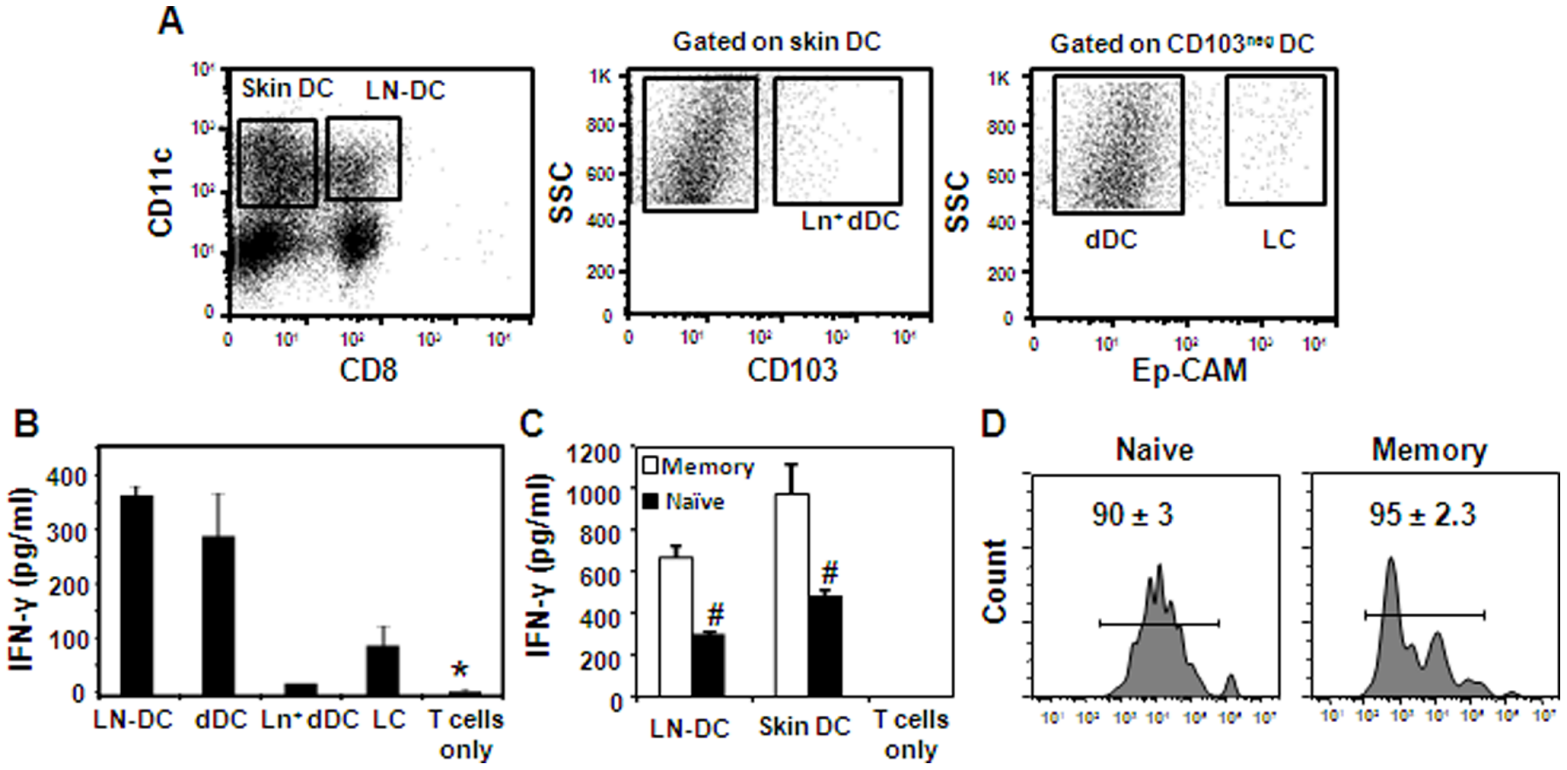
The contribution of DC subsets to antigen presenting activity after immunization with Lv-OVA. B6 mice were primed and boosted intradermally with Lv-OVA in the ear pinna. (**A**) Three days after the boosting immunization the draining LNs were pooled from 10 mice, CD11c^+^ population was enriched and the cells were further FACS-sorted according to the expression of the CD11c, CD103 and Ep-CAM molecules in the CD8^negative^ populations as described. (**B**) The various DC subsets were immediately incubated with purified OT-I CD8^+^ T cells and supernatants were collected 60 hr later to quantify the concentration of INF-γ by ELISA. (**C**) To analyze the capability of skin-derived DCs to activate memory and naïve CD8^+^ T cells, skin DCs (CD11c^+^CD8^neg^) and LN-DCs (CD11c^+^CD8^+^) were purified and incubated with naïve or memory OT-I CD8^+^ T cells generated *in vitro* using the SIINFEKL peptide. (**D**) Mice were primed with Lv-OVA and 24 hr later were administered with *in vitro*-generated memory CD8^+^ OT-I cells or naïve OT-I cells (1×10^6^ cells per mouse). Four days later, LNs were collected, processed and cell proliferation was analyzed. FACS plots are exhibited representing the mean of 3 mice per group ± SE. Results in this figure are representative of at least 2 independent experiments. *, *P*<0.05, IFN-γ secretion by T cells alone in comparison to T cells with DCs. #, *P*<0.01, IFN-γ secretion by memory T cells versus naïve cells.

The type of DC subsets involved in T-cell activation has been proposed to have a major influence on secondary CD8^+^ T-cell expansion [13]. To examine the role of DCs during secondary immunization with lentivectors, we collected lymph nodes (LNs) three days post-immunization with Lv-OVA, enriched CD11c^+^ cells and stained them with anti-CD11c, CD8, CD103 and Ep-CAM antibodies. The cells were then FACS-sorted to purify various DC populations according to the gating strategy illustrated in Fig. 3A. The purified DCs were co-cultured with OT-I cells for 60 hrs and IFN-γ secretion by the T cells was measured as an indication of their activation by antigen-bearing DCs. Fig. 3B demonstrated that secondary presentation of lentiviral-encoded antigen was mediated chiefly by LN-resident DCs and dermal DCs. In addition, lower presentation was found by Langerhans cells (LCs) while langerin-expressing CD103^+^ dermal DCs (Ln^+^dDCs) did not contribute to this process. Antigen presentation by LCs, however, seems to be negligible for T-cell priming *in vivo* (Fig. S3). Incubation of DCs from non-immunized mice with OT-I CD8^+^ T cells failed to induce significant IFN-γ secretion (data not shown). Next, we tested the capability of skin-derived DCs to present antigen to memory T cells, as a previous study suggested that diminished secondary CD8^+^ T-cell responses could be a result of minimal activation of memory cells by tissue-derived DCs [13]. However, as depicted in Fig. 3C, skin-derived DCs were able to efficiently stimulate *in vitro*-primed memory CD8^+^ OT-I T cells. To further confirm this finding *in vivo*, we administered *in vitro*-primed memory CD8^+^ OT-I T cells or control naïve cells into Lv-OVA primed mice. Note that Lv-OVA primed mice rather than boosted mice were employed to allow correct comparison of antigen presentation by DCs as previously reported [13], [14]. Four days later, LNs were collected and transferred T-cell proliferation was analyzed (Fig. 3D). As expected, memory and naïve T cells proliferated equally indicating that both CD8^+^ T cell types received similar levels of stimulation by DCs *in vivo*. Collectively, our data suggest that both LN-resident and tissue-derived DCs mediate antigen presentation to CD8^+^ T cells during secondary immunization with lentivectors. Furthermore, the diminished T-cell responses observed in boosted mice are probably not because of the inability of skin DCs to stimulate memory CD8^+^ T cells.

### CD8^+^ T-cell Responses Induced by Lentivectors Depend on Prolonged Antigen Expression

**Figure 4:**
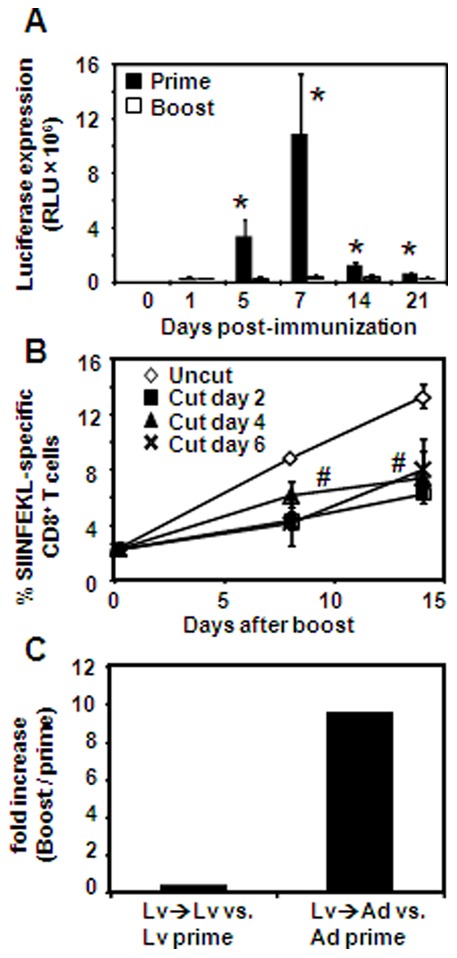
Kinetics of antigen expression control lentivector-induced secondary CD8^+^ T-cell response. (**A**) B6 mice were primed, or primed and boosted intradermally with 5×10^6^ TU of Lv-OVA/Luc, and luciferase expression was determined *in vivo* using whole body imagining. The mean relative light unit (RLU) values expressed by a group of 4 mice ± SE are presented. (**B**) Immunization site of Lv-OVA (5×10^6^ TU) homologously boosted mice was removed on days 2, 4 or 6 following immunization, and the magnitude of OVA-specific CD8^+^ T cells was analyzed. Data are showed as the mean percentage ± SE of CD8^+^ tetramer^+^ T cells. n  = 3 mice per group for each time point. (**C**) Mice were primed with Lv-OVA and 7 weeks later were boosted with Lv-OVA (Lv-Lv) or Ad-OVA (Lv-Ad). In parallel, other groups of naïve mice were primed with Lv-OVA (Lv prime) or Ad-OVA (Ad prime) using the same viral vector employed for boosting to allow adequate comparison. Two weeks after immunization tetramer analysis was performed on blood samples obtained from the mice (n  = 4 to 5 mice for each group). The graph represents the fold increase in the magnitude of OVA-specific CD8^+^ T cells, Lv-Lv versus Lv prime, and Lv-Ad versus Ad prime. Results described in this figure are representative of two independent experiments. *, *P*<0.01, prime versus boost response at the indicated time points. #, *P*<0.05, compared to control uncut boosted group.

In attempt to understand the brief period of antigen presenting activity during secondary immunization with lentivectors, we sought to characterize the kinetics of antigen expression induced by this vaccine modality. We immunized mice with Lv-OVA/Luc vector encoding both the OVA and luciferase gene (Fig. S1), and monitored luciferase expression *in vivo*. Low expression levels of luciferase were found in both primed and boosted mice until day 5 post-immunization (Fig. 4A). However, luciferase expression increased substantially in primed mice whereas in boosted mice the expression remained low until being cleared. We then asked whether the low expression levels measured after boosting immunization have any influence on CD8^+^ T-cell induction. For this, we primed and boosted mice with Lv-OVA and at days 2, 4 and 6 post boosting immunization, the ear pinna was excised or left intact. Tetramer analysis revealed that secondary expansion of OVA-specific CD8^+^ T cells was significantly reduced due to the excision of the immunization site (Fig. 4B). This suggests that even low levels of antigen expression is still presented by DCs after boosting and have an impact on T-cell activation; in agreement with the results presented in Fig. 2A, demonstrating the importance of prolonged secondary antigen expression for immune induction. We next examined if memory CD8^+^ T cells generated by lentivectors can be efficiently boosted, by immunizing Lv-primed mice with adenovector expressing OVA (Ad-OVA). A sub-optimal dose of Ad-OVA was chosen (10^6^ particles) to facilitate discrimination between the priming and boosting efficiencies of this vector. As illustrated in Fig. 4C, boosting lentivector-primed mice with adenovector (Lv/Ad) resulted in about a 9 fold increase in the frequencies of CD8^+^ T cells elicited by adenovector priming (Lv prime), whereas boosting with lentivectors (Lv/Lv) failed to expend primary responses induced by lentivectors (Lv prime). Taking together, these findings suggest that secondary immunization with lentivectors depends on prolonged antigen expression. However, lentivectors induced a unique slow kinetics of antigen expression, which in contrast to priming immunization failed to increase and might limit T-cell activation.

### The Gradual Increase in Antigen Load is not due to a Proliferation of Lentivector Transduced Cells

**Figure 5:**
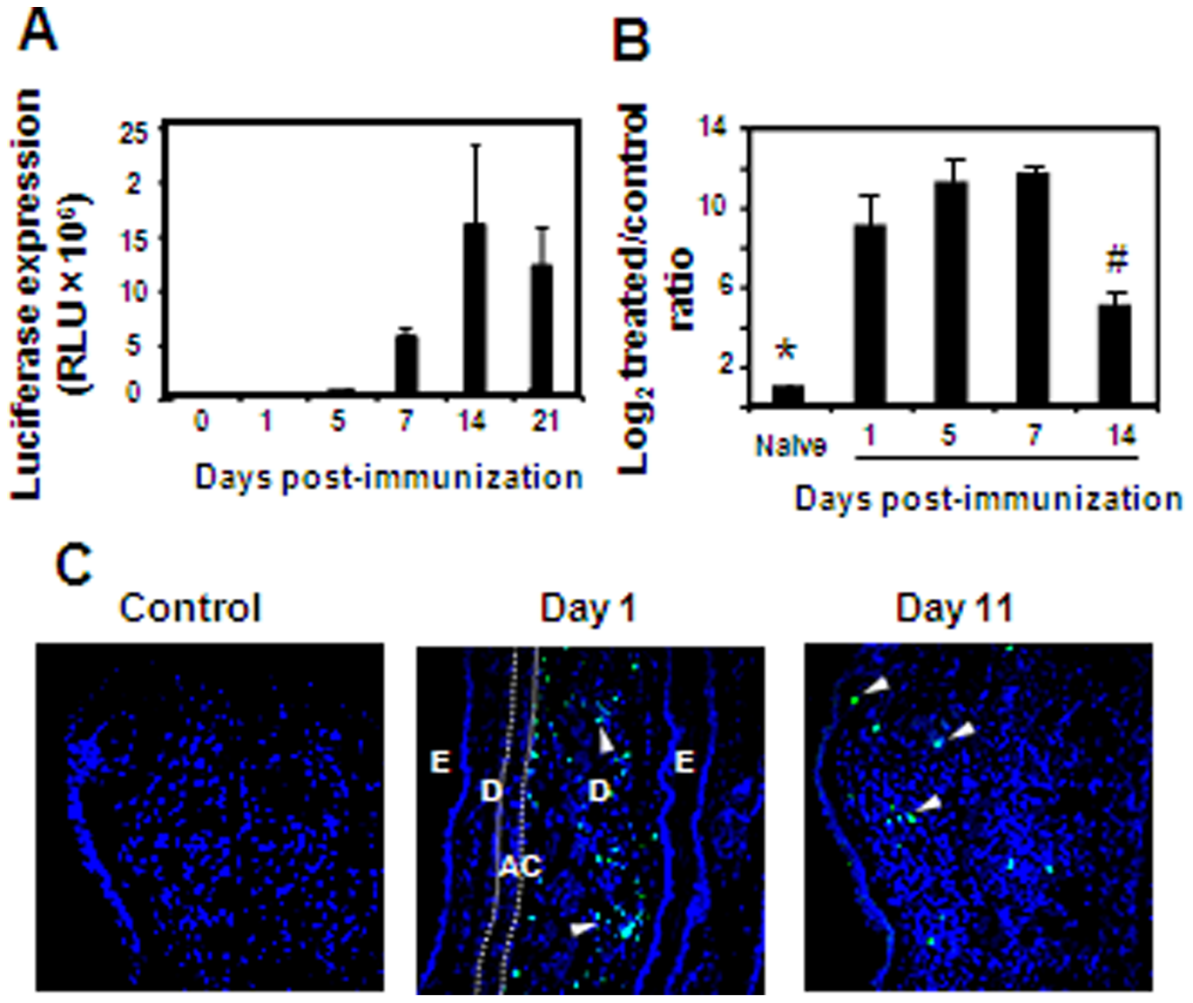
Lentivectors induce gradual increase in antigen load in expressor cells. (**A**) Mice were immunized with Lv-Luc vector (encoding also eGFP) and luciferase expression was tracked *in vivo*. (**B**) Part of the Lv-Luc primed mice were euthanized on days 1, 5, 7 and 14 and their ears were collected and processed to obtain genomic DNA. DNA samples were subjected to quantitative real-time PCR analysis in order to calculate the relative amount of lentiviral DNA in each day tested. Lentiviral DNA was quantified using eGFP specific primers and was standardized according to the levels of endogenous 18S DNA. (**C**) The ears of Lv-Luc immunized mice were removed on days 1 and 11 post priming and were then subjected to immunofluorescence analysis. Images of confocal microscopy of the ear pinna are shown with a 5-µm-thick section using a 10×0.6 objective and 25× zoom. Control image represents staining with secondary antibody only. (Blue, nuclei stained with the DNA intercalating dye DAPI; green, anti-GFP antibody). Arrow heads indicate GFP-expressing cells. Dotted line was added to define the auricular cartilage (AC), D-dermis, E-epidermis. One representative out of two independent experiments is depicted. *, *P*<0.005, compared to all immunized groups. #, *P*<0.001, compared to DNA copies measured on days 1, 5 and 7 post-immunization.

To further understand the unique expression kinetics of lentivectors encoded antigen *in vivo*, we characterized this process during priming immunization. We asked whether the elevated amounts of antigen observed overtime results from proliferation of lentivirally transduced cells which amplify the copies of integrated lentiviral genomes in the mice. To address this issue, we immunized intradermally a large cohort of mice with Lv-Luc (encoding both luciferase and eGFP), and monitored luciferase expression on days 1, 5, 7, 14 and 21 post-immunization (Fig. 5A). In parallel, the ears of part of the mice were collected, and total DNA was purified from them in order to calculate the relative amount of lentiviral DNA by real-time PCR using eGFP specific primers. The analysis indicated that a similar quantity of lentiviral genomic DNA was present in the immunization site from days 1 to 7 post-immunization (Fig. 5B). Of note, significant reduction in the number of lentiviral DNA copies was observed on day 14, the time in which antigen expression was maximal in the mice (Fig. 5A). To further demonstrate this point we performed an immunofluorescence staining on the ear skin of immunized mice (Fig. 5C). Higher numbers of eGFP-expressing cells were found in the ears of day 1 immunized mice as compared to ears taken on day 11 (133±32.5 versus 10.5±6.1 GFP-positive cells, receptively, *P*<0.005). These results thus suggest that the augmentation in antigen levels is not caused by an increase in lentiviral DNA copies as a result of proliferation of transduced cells.

### Rapid Antigen Clearance Diminishes Antigen Presentation Following Boosting with Lentivectors

**Figure 6:**
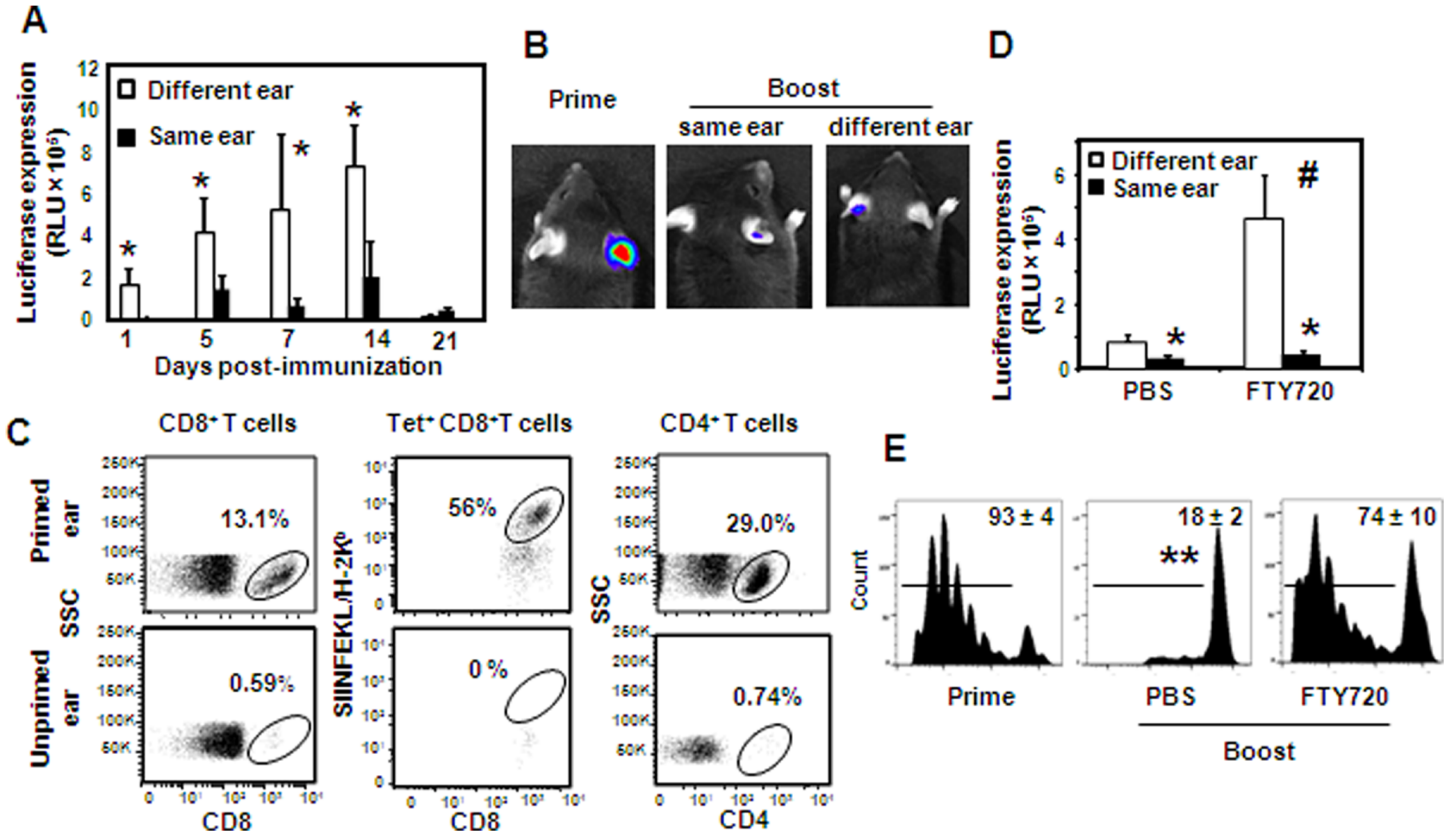
Blocking lymphocyte infiltration increased antigen expression and restored its presentation by DCs to CD8^+^ T cells. B6 mice were primed and boosted with Lv-Luc either at the same ear or at different ears. (**A**) Luciferase expression levels in the immunized mice presented as the mean RLU values expressed by a group of 4 mice ± SE. (**B**) Representative images of luciferase expression in Lv-Luc immunized mice 14 days following priming or boosting immunization in the same or different ear used for priming. (**C**) Representative flow plots display percentages of CD8^+^ tetramer^+^ T cells or CD4^+^ T cells in skin of immunized ear or the contralateral non-immunized ear 7 weeks after priming with Lv-OVA. (**D**) B6 mice were primed and boosted with Lv-OVA either at different ears or the same ear. The immunized mice were also administered with FTY720 (0.4 mg/ml per mouse) or PBS on a daily basis, starting on day −2 until day 5 of boosting immunization. On day 5 after boosting immunization the levels of luciferase expression in the ears of immunized mice (n = 4) either with or without FTY720 treatment were examined. (**E**) B6 mice were primed, or primed and boosted, with Lv-OVA at different ears. The boosted mice were administered with FTY720 or PBS as described. CFSE-labeled OT-I cells were adoptively transferred into the immunized mice 3 days after the immunization and LNs were collected 3 day later in order to analyze the dilution in CFSE levels. Results are shown as representative flow plots gating on dividing CD8^+^ T cells; numbers indicate the percentages of dividing cells and represent the mean of three mice per group ± SE. Results are representative of at least 2 independent experiments. *, *P*<0.05, luciferase levels in mice immunized in the same ear versus different ears. #, *P*<0.01, FTY720 versus PBS treatments. **, P<0.05, compared to CFSE dilution in primed mice or boosted mice with FTY720 treatment.

We demonstrated that the delayed kinetics of antigen expression induced by lentivectors allowed potent elicitation of primary T cell responses but not secondary responses. It is likely that T cells generated after priming rapidly clear secondary lentiviral-encoded antigen, preventing by that the typical gradual increase in its expression that might be critical for efficient immunogenicity. To address this issue we primed mice with Lv-Luc in the ear pinna and then boosted them either in the same or contralateral ear. Higher levels of luciferase expression were observed in mice boosted at different ears in comparison to boosting in the same ear (*P*<0.05 at each time point tested) (Fig. 6A). Still, in both groups the expression levels observed during the second week of immunization were drastically lower than that observed after priming (1.5 log reductions on day 14) (Fig. 6B and Fig. 4A). This suggests that in addition to local immune cells, circulating cells rapidly infiltrate the immunization site and clear antigen expression. To verify the presence of local antigen-specific immunity after priming, we analyzed the frequencies of T cells in Lv-OVA primed ears 7 weeks after immunization. As illustrated in Fig. 6C, the percentages of CD8^+^ T cells in the ear skin of primed mice were much higher than those in the contralateral unimmunized ear (13.1% vs. 0.59% respectively). More than 50% of these cells were specific to the OVA immunodominant MHC class I epitope SIINFEKL. Higher frequencies of CD4^+^ T cells were also found in the ear skin of immunized mice as compared to the unimmunized ear (29% vs. 0.74% respectively), further supporting the presence of robust local immunity.

We next examined the role of circulating lymphocytes in clearing secondary lentiviral-encoded antigens. We used temporal FTY720 (Fingolimod) treatment, which has been shown to block T-cell trafficking from both lymphoid organs and non-lymphoid tissues [15]. This treatment allowed us to prevent an infiltration of T cells from the circulation to the site of immunization. As demonstrated in Fig. S4, administration of FTY720 into boosted mice during the first 5 days of immunization reduced substantially the frequencies of CD3^+^ T lymphocytes in the blood in comparison to PBS treatment, consistent with an efficacious treatment (day 5 of immunization, *P* = 0.03). As a result, antigen expression increased in mice primed and boosted with Lv-OVA at different ears, whereas no effect was shown when the immunizations were given in the same ear (Fig. 6D). We then examined if the increase in antigen expression will restore the capability of DCs to activate memory CD8^+^ T cells. For this propose we transferred CFSE-labeled CD8^+^ OT-I cells into mice primed and boosted with Lv-OVA at different ears following FTY720 treatment. As demonstrated in Fig. 6E, the increase in antigen expression induced by FTY720 was able to restore efficiently antigen presentation to CD8^+^ T cells, though not the levels observed in primed mice. Altogether, our findings suggest that primary immune responses swiftly clear expression of lentivector-expressed secondary antigen. Since optimal CD8^+^ T-cell responses elicited by lentivectors relay on prolonged antigen expression, this activity considerably dampens secondary T-cell responses.

## Discussion

The failure of viral vectors to efficiently boost T cells in a setting of homologous prime-boost immunization is commonly explained by the presence of an anti-vector immunity. Here we provided a novel explanation for the inability of lentivectors, a very potent priming vector, to boost a potent cellular immunity. The delayed kinetics of antigen expression induced by lentivectors was shown to be a major pitfall during boosting, as it resulted in diminished levels of antigenic stimulation to memory T cells. Our observations that lentivectors failed to expand memory CD8^+^ T cells elicited by plasmid DNA (i.e. in the absence of any vector-specific immune responses), or following alteration of the envelope protein, further demonstrate the critical impact of the slow antigen expression on this process. Slow kinetics of transgene expression were also reported in other systems employing lentivectors [16], [17]. Integration of lentivectors into the genome of transduced cells is thought to be completed within hours and thus cannot explain this phenomenon. Of note, the gradual increase in antigen expression seen in our system is probably not due to proliferation of transduced cells which amplify lentiviral DNA. On the contrary, the levels of lentiviral DNA or lentiviral transduced cells actually decreased over time. It is thus likely that the augmentation in the amount of antigen we observed stems from an accumulation of the antigen within cells, or alternatively, results from an increase in expression level per cell.

It has been proposed that poor secondary immune responses could result from a preferential stimulation of naïve rather than memory CD8^+^ T cells by tissue-resident DCs [13]. This, however, seems not to be the case with lentivectors, as superior stimulation of memory cells, in comparison to naive cells, was mediated by skin-derived DCs *ex vivo* and *in vivo*; concurring with the common view that memory cells have less-stringent requirements for activation [18], [19]. It can also be ruled out that lentivectors failed to expand memory cells due to an intrinsic problem in these cells. As we and other demonstrated, memory CD8^+^ T cells generated after priming with lentivectors can be efficiently boosted with adenovectors or vaccinia [20]. In agreement, blocking lymphocytes infiltration and subsequently antigen clearance by FTY720, restored antigen expression and T-cell activation by DCs, indicating that antigen load is the major limitation during secondary immunization. We previously demonstrated that this antigen clearance is mediated by CD8^+^ T cells in a perforin-mediated pathway rather than a FAS-FASL signaling [21]. Nevertheless, the FTY720 treatment did not succeed to fully restore the proliferation levels of CD8^+^ T cells in comparison to primed mice (Fig. 6E). This could be explained by an incomplete blocking of T-cell infiltration into the immunized skin by the FTY720 treatment. Alternatively, in contrast to primed mice, endogenous memory CD8^+^ T cells present in the LNs of after priming are also capable of interacting with DCs after boosting, thus limiting the stimulation of the transferred CFSE-labeled cells.

The contribution of antigen persistence to the kinetics of a CD8^+^ T cell response seems to depend on the nature of the vaccine modality. A brief exposure to antigen was suggested to drive the clonal expansion of CD8^+^ T cells and their differentiation into memory cells [22], [23]. However, in some systems a prolonged antigen expression is required for optimal activation of CD8^+^ T cells. We have previously shown the importance of antigen persistence following intradermal plasmid DNA immunization [4]. Lentivectors also require durable antigen expression in order to prime CD8^+^ T cells, although to a lesser extent then plasmid DNA [21]. It is likely that the low expression levels induced by lentivectors on the first 5 days post-immunization are not capable to provide maximal stimulation to naïve T cells. As demonstrated in the present study, such kinetics of antigen expression are deleterious for secondary expansion of T cells.

The elicitation of secondary T-cell responses might be influenced by various immunological parameters. CD4-help, for instance, is critical during secondary immunization and lentivectors relay on such help to boost CD8^+^ T cells [20]. Our data is in line with this notion, as CD4^+^ T-cell responses were also impaired due to the slow kinetics of antigen expression. In addition, inflammatory signals and cytokines milieu induced by secondary immunization have a great impact on DC maturation and subsequently activation of T cells [24]. Thus one may suggest that secondary immunization with lentivectors does not facilitate DC maturation in comparison to priming immunization. However, we clearly demonstrated that activation of T cells was similar between days 0–3, a time in which comparable level of antigen was expressed in primed and boosted mice. Furthermore, DCs were able to present antigen efficiently when antigen expression was restored by the FTY720 treatment. These indicate that antigen load rather than stimulatory signals is the reason for the lower ability of DCs to activate T cells beyond day 3 after boosting. Finally, since OT-I CD8^+^ T cells were employed to monitor T-cell activation in primed and boosted mice; differences in TCR affinity can be ruled out as a possible explanation for the poor expansion of secondary T cells.

Previous studies have shown the capacity of lentivectors to boost memory CD8^+^ T cells following homologues or heterologous prime-boost immunization [25], [26], [27]. Nevertheless, in most of these works the magnitude of secondary T-cell responses was comparable or lower than those achieved during lentiviral priming. In fact, the reported enhancement in secondary immunity was referred to T-cell responses present in the mice at the time of boosting immunization. Interestingly, by priming and boosting mice with lentivectors engineered to target DCs *in vivo*, Dai et al. generated statistically significant higher secondary CD8^+^ T-cell responses than those measured in primed mice [28]. Although the levels of secondary tetramer-specific CD8^+^ T cells generated by this strategy were moderate as compared to other boosting vectors [4], it suggests that the boosting potential of lentivectors could be enhanced by efficient targeting of the antigen to DCs as previously suggested [29].

Accumulating data suggests that the absolute numbers of antigen-specific CD8^+^ T cells correlate linearly with their capacity to confer protection [30]. In order to increase cellular immunity over certain thresholds that are required for protection, repeated immunizations are obligatory [31]. Developing new approaches to accelerate the kinetics of antigen expression by lentivectors will facilitate the use of this attractive vaccine modality as a boosting agent in order to potentiate protective immunity.

## Materials and Methods

### Ethics Statement

Animal work was approved by the Hebrew University Institutional Animal Care and Ethic Committee (MD-09-12271-3).

### Antibodies and Reagents

The following monoclonal antibodies were purchased from BioLegends (San Diego, CA, USA) and used in the study: anti-CD8α (53–6.7), anti-CD103 (2E7), anti-CD11c (HL3), anti-CD4 (GK1.5) and anti-Ep-CAM (G8.8). SIINFEKL H-2K^b^ tetramers were purchased from Beckman Coulter (Brea, CA, USA). CFSE was purchased from Molecular Probes (Invitrogen, Grand Island, NY, USA) and FTY720 from Cayman Chemical (Ann Arbor, MI, USA).

### Construction and Production of Dual Promoter Lentivectors

Lentiviral constructs utilized the third generation, self-inactivating, replication incompetent lentiviral backbone vector, modified for dual promoter-dual transgene expression as we published previously [32], [33]. Construction, production and titration of the various lentivectors employed in the study were previously reported [21]. Briefly, a lentiviral construct for simultaneous dual transgene expression of ovalbumin (OVA) and eGFP (enhanced green fluorescence protein) cDNAs (CMV-OVA-UBC-eGFP; hereafter Lv-OVA; Fig. S1) was generated by 5′ *Not*I and 3′ *BamH*I ligation of the OVA cDNA into gene expression position 1 (downstream of the CMV promoter), and ligation of the eGFP (Clontech) cDNA into gene expression position 2 (downstream of the human ubiquitin-C (UBC) promoter). An additional lentiviral construct for dual transgene expression of both luciferase and eGFP cDNAs (CMV-Luc-UBC-eGFP; hereafter Lv-Luc; Fig. S1) was generated by similar 5′*Not*I/3′*BamH*I ligation of the cDNA encoding firefly luciferase into gene expression position 1. Vesicular stomatitis virus glycoprotein (VSV-G) pseudotyped lentivirus was generated by triple transfection of 293T cells with the lentiviral backbone construct together with two helper plasmids encoding the viral genes Gag-Pol-Tat-Rev, and VSV-G. In some experiments plasmid encoding the VSV-G gene was replaced by plasmid encoding the envelope gene of the amphotropic murine leukemia virus (Ampho) (a kind gift from Prof. Amos Panet, the Hebrew University, Jerusalem). Cell supernatants containing virus were concentrated by centrifugation (90 min; 48,960×g). Titers of eGFP-expressing lentiviruses were calculated as “293-transducing units” per ml (TU/ml) based on flow cytometry of infected 293T cells, and concentrated titers of 2×10^8^ TU/mL were employed in all experiments. Titer of Lv-OVA-Luc lentivirus was estimated using Leni-X GoStix (clontech) and was compared to a known concentration of eGFP-expressing lentivirus.

### Mice

Six- to eight-week-old C57BL/6 (B6), OT-I and OT-II mice were purchased from the Jackson laboratories (Bar Harbor, ME, USA). OT-I mice carry a transgenic CD8 T-cell receptor (TCR) for the MHC class I–restricted OVA_257–264_ peptide; OT-II mice carry a transgenic CD4 TCR specific for the MHC class II-restricted OVA_323–339_ peptide.

### Immunizations and Challenge

Mice were anaesthetized with ketamine/xylazine mix and a 31 gauge needle was used to inject intradermally 5×10^6^ TU (transduction units) of lentivectors per mouse. Similar procedure was adopted for injecting recombinant adenovectors type 5 encoding OVA (Ad-OVA) (10^6^ or 5×10^7^ particles), which were kindly provided by Dr. Norm Letvin (Harvard University, MA, USA). The pACB-OVA plasmid DNA (a gift from Dr. Maripat Corr, UCSD) was given intradermally, 50 µg of DNA in 80 µl total injection volume (40 µl was delivered into each ear). Seven weeks after the first immunization, mice were boosted, either homologously or heterologously, via the same route and quantity as described for the priming immunization. Tumor challenge was performed by injecting a lethal dose of B16-OVA cells (1×10^6^ cells per mouse) subcutaneously to the flank 15 days after immunization. Removal of the ear pinna was performed using sterile scissors either following anaesthetization with ketamine/xylazine mix or euthanization as indicated in the text.

### Tetramer Analysis

Blood was collected from individual mice in RPMI 1640 medium containing 40 U of heparin per ml. Red blood cells were lysed using ACK buffer and the samples were washed with PBS containing 2% fetal calf serum (FCS) and stained for 15 min at RT with H-2K^b^/SIINFEKL tetramers. The cells were then stained with anti-CD8α antibody for an additional 15 min at RT, washed with PBS containing 2% FCS. Samples were collected on a LSR II instrument (BD Biosciences, San Jose, CA, USA) and analyzed using the FlowJo software (Tree Star, Ashland, OR, USA).

### Bioimaging of Luciferase Protein Expression

Whole body imaging of *in vivo* firefly luciferase gene expression was performed using the IVIS® Kinetic instrument (Caliper Life Sciences, MA, USA). Mice were anaesthetized with ketamine/xylazine mix and injected intraperitoneally with 500 µl of an isotonic salt solution containing 30 mg/ml D-Luciferin. Twenty minutes after luciferin injection, photonic emissions were measured and raw data were analyzed using Living Image 4.0 software to assess photon flux in regions of interest in each mouse.

### Generation of Memory CD8^+^ T cells

The generation of in vitro-primed memory CD8^+^ T cells were done as described [13]. Briefly, naïve OT-I transgenic spleen cells were coated for 1 hr at 37°C with 1 µM SIINFEKL peptide. Cells were then washed twice with HEPES-buffered DMEM medium containing 2.5% (vol/vol) FCS before being cultured at a density of 2×10^5^ cells per ml in complete medium (mouse tonicity RPMI 1640 medium: RPMI 1640 medium containing 10% (vol/vol) FCS, 50 µM β-mercaptoethanol, 2 mM L-glutamine, 100 U/ml of penicillin and 100 µg/ml of streptomycin ('complete medium')). After 2 days, cells were washed and supplemented with recombinant human IL-15 (20 ng/ml; R&D Systems). Complete medium containing human IL-15 was replaced every 3–4 days, and cells were used 14 days after initiation of the culture.

### Blockage of Peripheral T-cell Recruitment

Starting on day -2 of boosting immunization, mice were injected i.p with 400 µl of 0.4 mg/ml FTY720 solution, and the treatment was proceed on a daily basis until day 5 of immunization. The efficacy of this treatment to block T-cell circulation was confirmed by measuring CD3-positive lymphocytes in the peripheral blood [15].

### *In vivo* T-cell Proliferation Assay

Splenocytes were obtained from OT-I or OT-II mice and washed with PBS. The cells were diluted in HBSS (4×10^6^ cells/ml) and then incubated with same volume of 5 µM CFSE in HBSS for 10 min at 37°C at a final concentration of 2.5 µM. Labeling was quenched by adding an excess of ice-cold RPMI 1640 complete medium and the cells were washed twice with PBS. CFSE-labeled splenocytes (2×10^6^) in 200 ml PBS were transferred into Lv-OVA primed or homogonously-boosted mice by i.v. tail injection. Three days following cell transfer, mice were sacrificed and the draining LNs were harvested. The level of CFSE dilution was determined by flow cytometry using anti-CD8α and anti-CD4 antibodies.

### Antigen Presentation Assays

Draining LNs were collected from immunized mice three days post primary or secondary immunization with Lv-OVA. In order to assess the capability of DCs to present antigen to memory versus naïve T cells, LNs were collected 4 days post-immunization. The LNs where then treated with collagenase type II (1 mg/ml, Worthington Biochemicals, Lakewood, NJ, USA) and DNase I (1 mg/ml, Roche, Hod Hasharon, Israel) solution in PBS +2% FCS for 20 min at 37°C in a shaker bath. 10 µl/ml of EDTA 0.5 M was added to the digested LNs and the incubation was continued for an additional 10 min. The cells were then washed and filtered. CD11c^+^ cells were obtained from the digested LNs by positive isolation using MACS Microbeads according to the manufacturer’s instructions (Miltenyi Biotec, Bergisch Gladbach, Germany). The enriched CD11c^+^ cells were stained with antibodies against CD103, CD8α, CD11c and Ep-CAM and then subjected to sorting by flow cytometry (FACSAria). OT-I CD8^+^ T cells were purified by negative selection with the EasyStep mouse CD8^+^ T cell enrichment kit according to the manufacturer’s instructions (StemCell Technologies, Vancouver, British Columbia, Canada). The purified OT-I CD8^+^ T cells (5×10^4^/well) were incubated with each indicated DC population (3×10^4^/well) in 96 well U-Plates (Nunc, Rosklide, Denmark). The cells were then incubated for 60 hr and the IFN-γ levels were measured in the supernatant of T cell-DC cultures, using the ELISA MAX™ mouse IFN-γ kit (BioLegend, San Diego, CA, USA) according to the manufacturer's instructions. Cytokine levels were determined using standard curves of recombinant IFN-γ cytokine and are expressed as picograms per milliliter.

### Isolation of Lymphocytes from Skin Tissues

The ear pinna of naïve or immunized mice were excised, washed with 70% ethanol for 1 min and then with PBS. The skin was separated into two halves, minced to little pieces and incubated for 30 min at 37°C with collagenase/DNase solution (1 mg/ml). EDTA 0.5 M was added to the digested skin and the incubation was continued for an additional 10 min. The cells were washed with PBS containing 2% FCS and stained for 15 min at RT with H-2K^b^/SIINFEKL tetramers. The cells were then stained with anti-CD8α or CD4 antibodies for an additional 15 min at RT, washed with PBS containing 2% FCS.

### Real-Time PCR Analysis

Mice were immunized with Lv-Luc and on days 1, 7 and 14 of immunization mice were euthanized and the injected ears were immediately collected. The ears were treated overnight with Proteinase K (Sigma, Rehovot, Israel) followed by isopropanol precipitation to elute genomic DNA. In order to quantify the amount of lentiviral genomic DNA, the Primer Express software (Applied Biosystems, Carlsbad, CA, USA) was employed to generate the following primers against eGFP and the endogenous mouse ribosomal 18S genes: eGFP-F: 5′-GGGCACAAGCTGGAGTACAACT-3′, eGFP-R 5′-ATGTTGTGGCGGATCTTG AAGT-3′, mouse 18S-F: 5′-CGGCTACCACATCCAAGGAA-3′, 18S-R 5′-GG GCCTCGAAAGAGTCCTGTAT -3′. Reactions were performed in an ABI Prism 7700 System (Applied Biosystems, Carlsbad, CA, USA), in a 20 µl reaction volume containing 10 µl of SYBR Green Master Mix (Invitrogen, Grand Island, NY, USA), 500 nM of each forward and reverse primer, and 5 µl of diluted DNA. The appropriate DNA dilution was calibrated for each primer couple. The thermal profile for SYBR Green RT-PCR was 95°C for 10 min, followed by 40 cycles of 95°C for 15 s and 60°C for 1 min. Analysis of the results was performed by the ΔCt method, which reflects the difference in threshold for the target gene relative to that of mouse 18S in each sample.

### Immunofluorescence Staining

The ears of lentivector-immunized and naive mice were excised, formalin fixed and paraffin embedded. Tissue sections of 5 µM were deparaffinized in xylene and rehydrated in decreasing concentrations of ethanol (100, 96, and 80%). Antigen retrieval was done for 2.45 min at 125°C in citrate buffer (pH 6.0). After cooling, the slides were washed with PBS and blocked with CAS Block buffer (Invitrogen) for 20 min. Next, the blocking buffer was replaced with CAS Block buffer containing the primary antibody mouse anti-GFP (Invitrogen; 33–2600) at a 1/100 dilution. Slides remained in the humidified chamber and were incubated at 4°C overnight. The slides were washed three times with PBS and a secondary antibody Alexa 488-conjugated donkey anti-mouse IgG (Jackson) was applied for 30 min at RT at a 1/200 dilution. The samples were washed three times in PBS and counterstained with DAPI (MP Biomedicals, Solon, OH; 157574) solution (1 mg/ml PBS) for 30 min at RT in the dark. The samples were then washed twice in tap water and mounted with fluorescent mounting medium (Dako, Glostrup, Denmark; S3623). Images were obtained using a Zeiss LSM 710 Axio observer.Z1 with an EC PlnN 10×0.6 lens and 25 zoom.

### Statistical Analysis

Data were expressed as means ± standard error of the means (SE). Statistical tests were performed using one-way analysis of variance (ANOVA) and the Student’s *t*-test. Kaplan-Meier survival curve and logrank test was performed using the Prism 4 software (GraphPad Software Inc.). *P* value <0.05 was considered significant.

## Supporting Information

Figure S1 Schematic illustration of lentiviral vectors employed in the study.
(PPTX)

Figure S2 Lentivector-elicited CD8^+^ T cells differentiate mainly into effector-memory cells.
(PPTX)

Figure S3 Langerin-expressing cells are dispensable for lentivector-induced secondary CD8^+^ T-cell responses.
(PPTX)

Figure S4 Blocking circulating lymphocytes by FTY720.
(PPTX)

## References

[1] Sallusto F, Lanzavecchia A, Araki K, Ahmed R (2010) From vaccines to memory and back. Immunity 33: 451–463. 21029957 10.1016/j.immuni.2010.10.008 PMC3760154

[2] Paris RM, Kim JH, Robb ML, Michael NL (2010) Prime-boost immunization with poxvirus or adenovirus vectors as a strategy to develop a protective vaccine for HIV-1. Expert Rev Vaccines 9: 1055–1069. 20822348 10.1586/erv.10.106

[3] Rollier CS, Reyes-Sandoval A, Cottingham MG, Ewer K, Hill AV (2011) Viral vectors as vaccine platforms: deployment in sight. Curr Opin Immunol 23: 377–382. 21514130 10.1016/j.coi.2011.03.006

[4] Hovav AH, Panas MW, Osuna CE, Cayabyab MJ, Autissier P, et al (2007) The impact of a boosting immunogen on the differentiation of secondary memory CD8+ T cells. J Virol 81: 12793–12802. 17881444 10.1128/JVI.01519-07 PMC2169130

[5] Kootstra NA, Verma IM (2003) Gene therapy with viral vectors. Annu Rev Pharmacol Toxicol 43: 413–439. 12359866 10.1146/annurev.pharmtox.43.100901.140257

[6] Beignon AS, Mollier K, Liard C, Coutant F, Munier S, et al (2009) Lentiviral vector-based prime/boost vaccination against AIDS: pilot study shows protection against Simian immunodeficiency virus SIVmac251 challenge in macaques. J Virol 83: 10963–10974. 19706700 10.1128/JVI.01284-09 PMC2772810

[7] Adotevi O, Mollier K, Neuveut C, Dosset M, Ravel P, et al (2010) Targeting human telomerase reverse transcriptase with recombinant lentivector is highly effective to stimulate antitumor CD8 T-cell immunity in vivo. Blood 115: 3025–3032. 20130242 10.1182/blood-2009-11-253641

[8] Iglesias MC, Frenkiel MP, Mollier K, Souque P, Despres P, et al (2006) A single immunization with a minute dose of a lentiviral vector-based vaccine is highly effective at eliciting protective humoral immunity against West Nile virus. J Gene Med 8: 265–274. 16308885 10.1002/jgm.837

[9] Liu Y, Peng Y, Mi M, Guevara-Patino J, Munn DH, et al (2009) Lentivector immunization stimulates potent CD8 T cell responses against melanoma self-antigen tyrosinase-related protein 1 and generates antitumor immunity in mice. J Immunol 182: 5960–5969. 19414747 10.4049/jimmunol.0900008 PMC3077746

[10] Santra S, Liao HX, Zhang R, Muldoon M, Watson S, et al (2010) Mosaic vaccines elicit CD8+ T lymphocyte responses that confer enhanced immune coverage of diverse HIV strains in monkeys. Nat Med 16: 324–328. 20173754 10.1038/nm.2108 PMC2834806

[11] Woodland DL (2004) Jump-starting the immune system: prime-boosting comes of age. Trends Immunol 25: 98–104. 15102369 10.1016/j.it.2003.11.009

[12] Ranasinghe C, Ramshaw IA (2009) Genetic heterologous prime-boost vaccination strategies for improved systemic and mucosal immunity. Expert Rev Vaccines 8: 1171–1181. 19722891 10.1586/erv.09.86

[13] Belz GT, Bedoui S, Kupresanin F, Carbone FR, Heath WR (2007) Minimal activation of memory CD8+ T cell by tissue-derived dendritic cells favors the stimulation of naive CD8+ T cells. Nat Immunol 8: 1060–1066. 17767161 10.1038/ni1505

[14] Bedoui S, Davey GM, Lew AM, Heath WR (2009) Equivalent stimulation of naive and memory CD8 T cells by DNA vaccination: a dendritic cell-dependent process. Immunol Cell Biol 87: 255–259. 19172155 10.1038/icb.2008.105

[15] Yopp AC, Ledgerwood LG, Ochando JC, Bromberg JS (2006) Sphingosine 1-phosphate receptor modulators: a new class of immunosuppressants. Clin Transplant 20: 788–795. 17100731 10.1111/j.1399-0012.2006.00570.x

[16] Fleury S, Simeoni E, Zuppinger C, Deglon N, von Segesser LK, et al (2003) Multiply attenuated, self-inactivating lentiviral vectors efficiently deliver and express genes for extended periods of time in adult rat cardiomyocytes in vivo. Circulation 107: 2375–2382. 12695294 10.1161/01.CIR.0000065598.46411.EF

[17] Parker DG, Kaufmann C, Brereton HM, Anson DS, Francis-Staite L, et al (2007) Lentivirus-mediated gene transfer to the rat, ovine and human cornea. Gene Ther 14: 760–767. 17301843 10.1038/sj.gt.3302921

[18] Carbone FR, Belz GT, Heath WR (2004) Transfer of antigen between migrating and lymph node-resident DCs in peripheral T-cell tolerance and immunity. Trends Immunol 25: 655–658. 15530835 10.1016/j.it.2004.09.013

[19] Croft M, Bradley LM, Swain SL (1994) Naive versus memory CD4 T cell response to antigen. Memory cells are less dependent on accessory cell costimulation and can respond to many antigen-presenting cell types including resting B cells. J Immunol 152: 2675–2685. 7908301

[20] Xiao H, Peng Y, Hong Y, Liu Y, Guo ZS, et al (2011) Lentivector prime and vaccinia virus vector boost generate high-quality CD8 memory T cells and prevent autochthonous mouse melanoma. J Immunol 187: 1788–1796. 21746967 10.4049/jimmunol.1101138 PMC3150273

[21] Furmanov K, Elnekave M, Lehmann D, Clausen BE, Kotton DN, et al (2010) The role of skin-derived dendritic cells in CD8+ T cell priming following immunization with lentivectors. J Immunol 184: 4889–4897. 20357252 10.4049/jimmunol.0903062

[22] Mercado R, Vijh S, Allen SE, Kerksiek K, Pilip IM, et al (2000) Early programming of T cell populations responding to bacterial infection. J Immunol 165: 6833–6839. 11120806 10.4049/jimmunol.165.12.6833

[23] Badovinac VP, Porter BB, Harty JT (2002) Programmed contraction of CD8(+) T cells after infection. Nat Immunol 3: 619–626. 12055624 10.1038/ni804

[24] Macagno A, Napolitani G, Lanzavecchia A, Sallusto F (2007) Duration, combination and timing: the signal integration model of dendritic cell activation. Trends Immunol 28: 227–233. 17403614 10.1016/j.it.2007.03.008

[25] Dullaers M, Van Meirvenne S, Heirman C, Straetman L, Bonehill A, et al (2006) Induction of effective therapeutic antitumor immunity by direct in vivo administration of lentiviral vectors. Gene Ther 13: 630–640. 16355115 10.1038/sj.gt.3302697

[26] Esslinger C, Chapatte L, Finke D, Miconnet I, Guillaume P, et al (2003) In vivo administration of a lentiviral vaccine targets DCs and induces efficient CD8(+) T cell responses. J Clin Invest 111: 1673–1681. 12782670 10.1172/JCI17098 PMC156105

[27] Iglesias MC, Mollier K, Beignon AS, Souque P, Adotevi O, et al (2007) Lentiviral vectors encoding HIV-1 polyepitopes induce broad CTL responses in vivo. Mol Ther 15: 1203–1210. 17375069 10.1038/sj.mt.6300135

[28] Dai B, Yang L, Yang H, Hu B, Baltimore D, et al (2009) HIV-1 Gag-specific immunity induced by a lentivector-based vaccine directed to dendritic cells. Proc Natl Acad Sci U S A 106: 20382–20387. 19918062 10.1073/pnas.0911742106 PMC2777969

[29] Cockburn IA, Chakravarty S, Overstreet MG, Garcia-Sastre A, Zavala F (2008) Memory CD8+ T cell responses expand when antigen presentation overcomes T cell self-regulation. J Immunol 180: 64–71. 18097005 10.4049/jimmunol.180.1.64

[30] Wherry EJ, Teichgraber V, Becker TC, Masopust D, Kaech SM, et al (2003) Lineage relationship and protective immunity of memory CD8 T cell subsets. Nat Immunol 4: 225–234. 12563257 10.1038/ni889

[31] Seder RA, Hill AV (2000) Vaccines against intracellular infections requiring cellular immunity. Nature 406: 793–798. 10963610 10.1038/35021239

[32] Wilson AA, Kwok LW, Hovav AH, Ohle SJ, Little FF, et al (2008) Sustained expression of alpha1-antitrypsin after transplantation of manipulated hematopoietic stem cells. Am J Respir Cell Mol Biol 39: 133–141. 18323534 10.1165/rcmb.2007-0133OC PMC2542452

[33] Wilson AA, Murphy GJ, Hamakawa H, Kwok LW, Srinivasan S, et al (2010) Amelioration of emphysema in mice through lentiviral transduction of long-lived pulmonary alveolar macrophages. J Clin Invest 120: 379–389. 20038801 10.1172/JCI36666 PMC2798672

