# Supplementary material for: Diminished Memory T-Cell Expansion Due to Delayed Kinetics of Antigen Expression by Lentivectors
Source: PLoS One. 2013 Jun 18;8(6):e66488. doi: 10.1371/journal.pone.0066488 (PMC3688922; doi:10.1371/journal.pone.0066488)
Supplement: Figure S1 — Schematic illustration of lentiviral vectors employed in the study. (PPTX) [file pone.0066488.s001.pptx]

## Slide 1
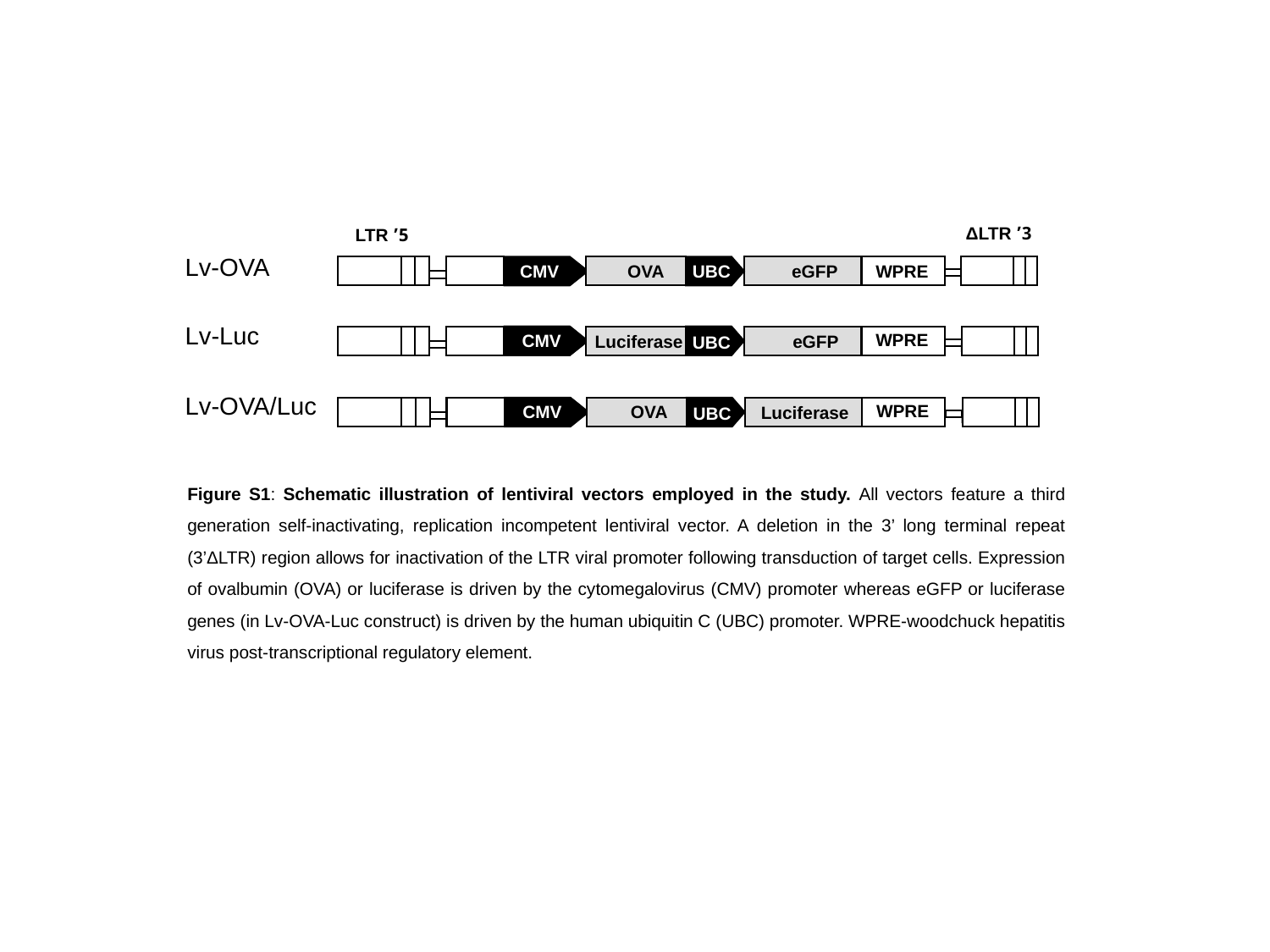

3’ ΔLTR
5’ LTR
Lv-OVA
CMV
OVA
eGFP
UBC
WPRE
Lv-Luc
WPRE
CMV
Luciferase
eGFP
UBC
Lv-OVA/Luc
WPRE
OVA
CMV
Luciferase
UBC
Figure S1: Schematic illustration of lentiviral vectors employed in the study. All vectors feature a third generation self-inactivating, replication incompetent lentiviral vector. A deletion in the 3’ long terminal repeat (3’ΔLTR) region allows for inactivation of the LTR viral promoter following transduction of target cells. Expression of ovalbumin (OVA) or luciferase is driven by the cytomegalovirus (CMV) promoter whereas eGFP or luciferase genes (in Lv-OVA-Luc construct) is driven by the human ubiquitin C (UBC) promoter. WPRE-woodchuck hepatitis virus post-transcriptional regulatory element.
