# Supplementary material for: Diminished Memory T-Cell Expansion Due to Delayed Kinetics of Antigen Expression by Lentivectors
Source: PLoS One. 2013 Jun 18;8(6):e66488. doi: 10.1371/journal.pone.0066488 (PMC3688922; doi:10.1371/journal.pone.0066488)
Supplement: Figure S2 — Lentivector-elicited CD8+ T cells differentiate mainly into effector-memory cells. (PPTX) [file pone.0066488.s002.pptx]

## Slide 1
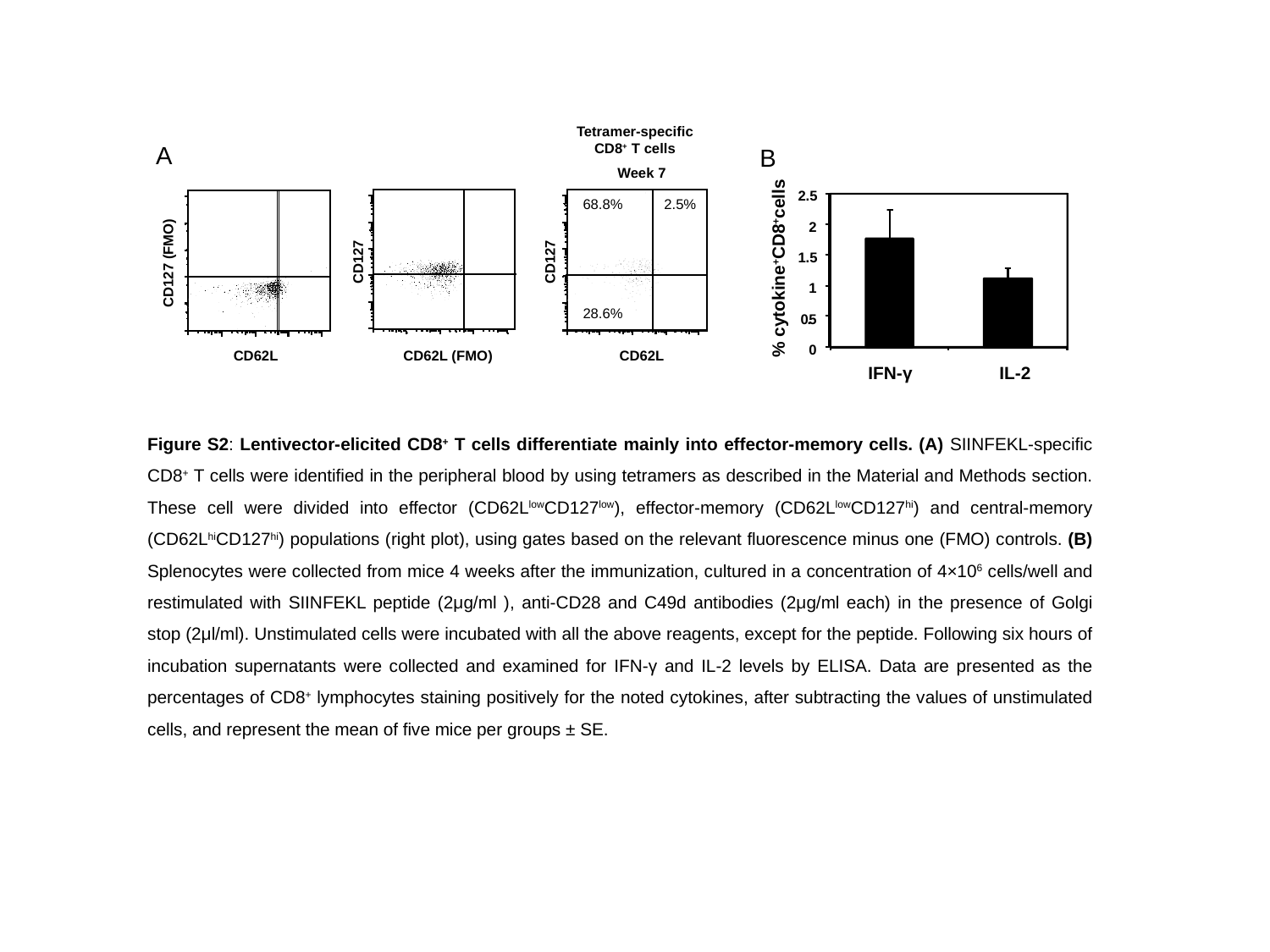

Tetramer-specific CD8+ T cells
Week 7
68.8%
2.5%
CD127
CD127 (FMO)
CD127
28.6%
CD62L
CD62L (FMO)
CD62L
A
 B
2.5
2
1.5
% cytokine+CD8+cells
1
0
.
5
0
IFN-γ
IL-2
Figure S2: Lentivector-elicited CD8+ T cells differentiate mainly into effector-memory cells. (A) SIINFEKL-specific CD8+ T cells were identified in the peripheral blood by using tetramers as described in the Material and Methods section. These cell were divided into effector (CD62LlowCD127low), effector-memory (CD62LlowCD127hi) and central-memory (CD62LhiCD127hi) populations (right plot), using gates based on the relevant fluorescence minus one (FMO) controls. (B) Splenocytes were collected from mice 4 weeks after the immunization, cultured in a concentration of 4×10^6^ cells/well and restimulated with SIINFEKL peptide (2μg/ml ), anti-CD28 and C49d antibodies (2μg/ml each) in the presence of Golgi stop (2μl/ml). Unstimulated cells were incubated with all the above reagents, except for the peptide. Following six hours of incubation supernatants were collected and examined for IFN-γ and IL-2 levels by ELISA. Data are presented as the percentages of CD8+ lymphocytes staining positively for the noted cytokines, after subtracting the values of unstimulated cells, and represent the mean of five mice per groups ± SE.
