# Supplementary material for: Diminished Memory T-Cell Expansion Due to Delayed Kinetics of Antigen Expression by Lentivectors
Source: PLoS One. 2013 Jun 18;8(6):e66488. doi: 10.1371/journal.pone.0066488 (PMC3688922; doi:10.1371/journal.pone.0066488)
Supplement: Figure S3 — Langerin-expressing cells are dispensable for lentivector-induced secondary CD8+ T-cell responses. (PPTX) [file pone.0066488.s003.pptx]

## Slide 1
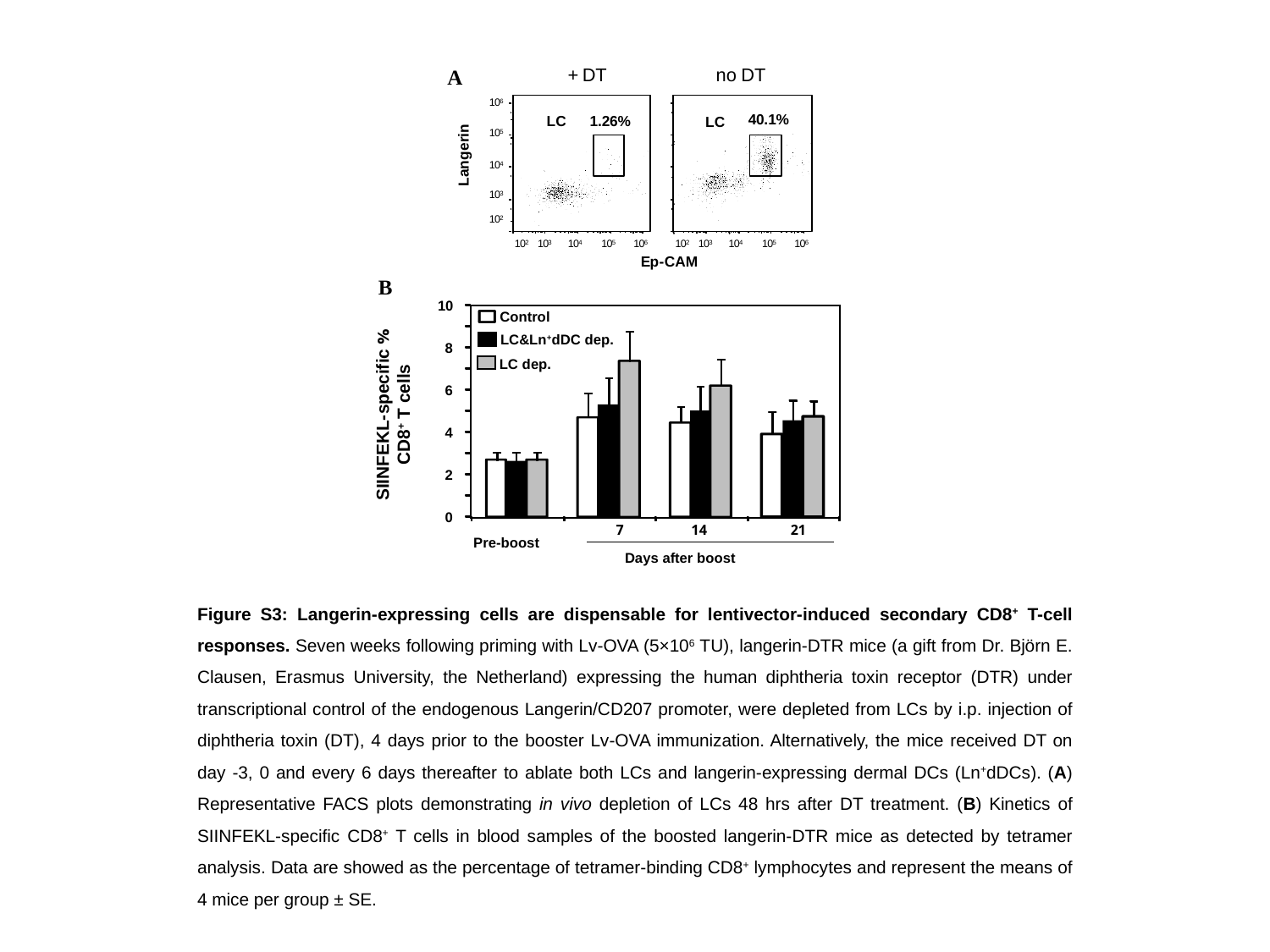

A
B
10
Control
LC&Ln+dDC dep.
LC dep.
8
6
% SIINFEKL-specific CD8+ T cells
4
2
0
7
14
21
Pre-boost
Days after boost
Figure S3: Langerin-expressing cells are dispensable for lentivector-induced secondary CD8+ T-cell responses. Seven weeks following priming with Lv-OVA (5×10^6^ TU), langerin-DTR mice (a gift from Dr. Björn E. Clausen, Erasmus University, the Netherland) expressing the human diphtheria toxin receptor (DTR) under transcriptional control of the endogenous Langerin/CD207 promoter, were depleted from LCs by i.p. injection of diphtheria toxin (DT), 4 days prior to the booster Lv-OVA immunization. Alternatively, the mice received DT on day -3, 0 and every 6 days thereafter to ablate both LCs and langerin-expressing dermal DCs (Ln+dDCs). (A) Representative FACS plots demonstrating in vivo depletion of LCs 48 hrs after DT treatment. (B) Kinetics of SIINFEKL-specific CD8+ T cells in blood samples of the boosted langerin-DTR mice as detected by tetramer analysis. Data are showed as the percentage of tetramer-binding CD8+ lymphocytes and represent the means of 4 mice per group ± SE.
