# Supplementary material for: Diminished Memory T-Cell Expansion Due to Delayed Kinetics of Antigen Expression by Lentivectors
Source: PLoS One. 2013 Jun 18;8(6):e66488. doi: 10.1371/journal.pone.0066488 (PMC3688922; doi:10.1371/journal.pone.0066488)
Supplement: Figure S4 — Blocking circulating lymphocytes by FTY720. (PPTX) [file pone.0066488.s004.pptx]

## Slide 1
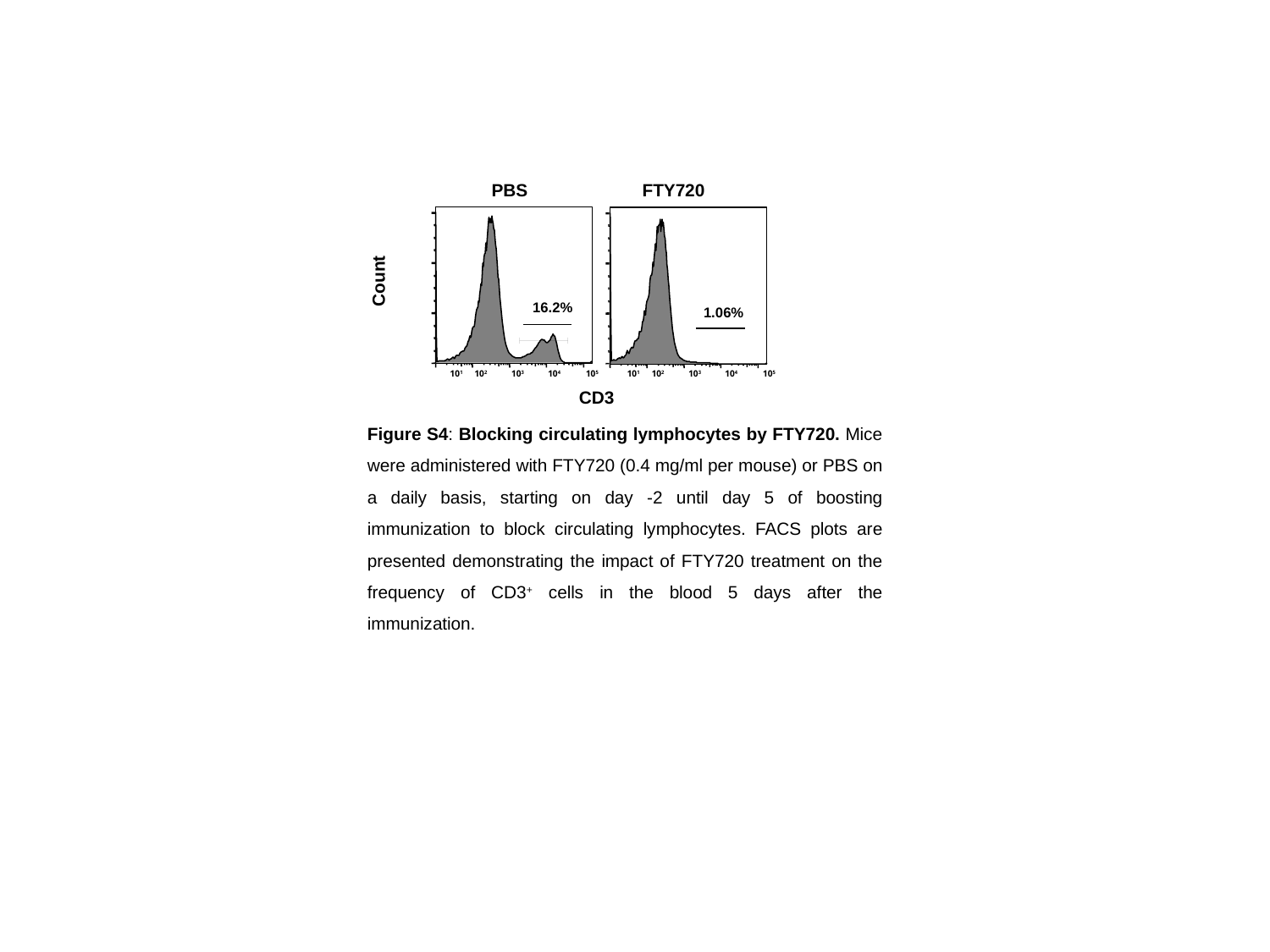

FTY720
1.06%
101
102
103
104
105
PBS
16.2%
Count
101
102
103
104
105
CD3
Figure S4: Blocking circulating lymphocytes by FTY720. Mice were administered with FTY720 (0.4 mg/ml per mouse) or PBS on a daily basis, starting on day -2 until day 5 of boosting immunization to block circulating lymphocytes. FACS plots are presented demonstrating the impact of FTY720 treatment on the frequency of CD3+ cells in the blood 5 days after the immunization.
